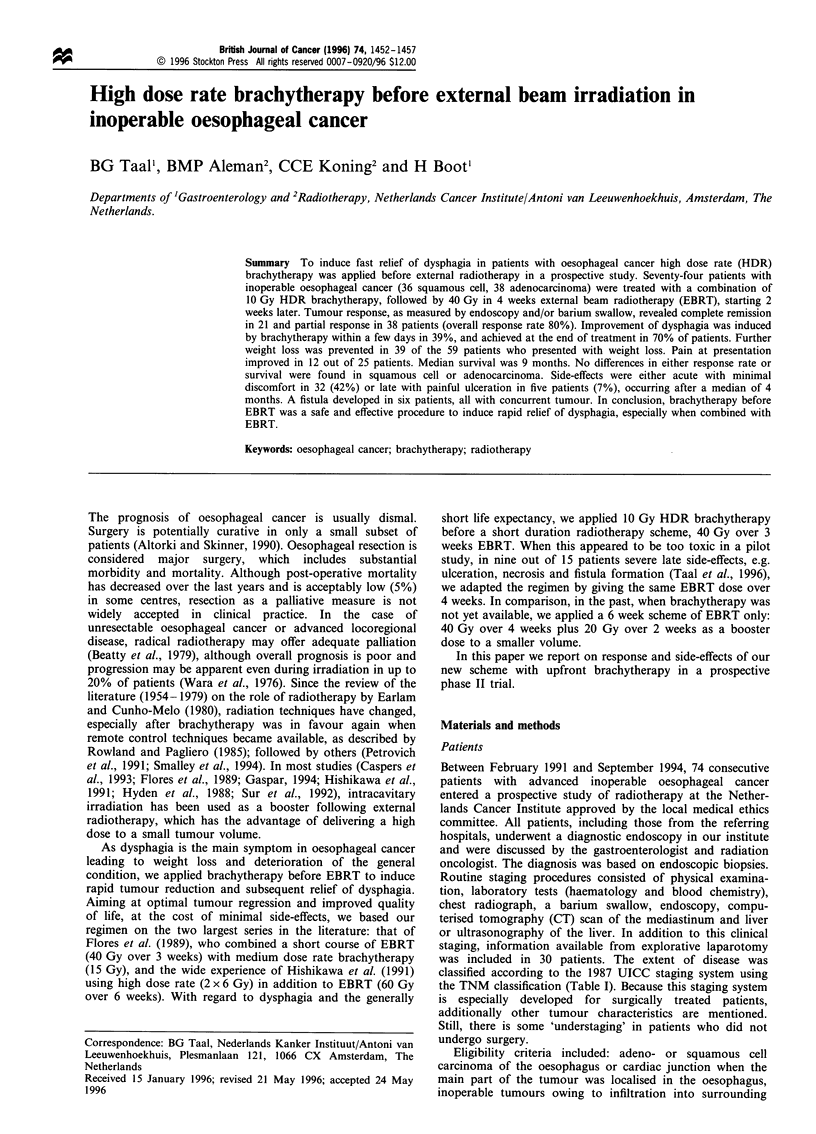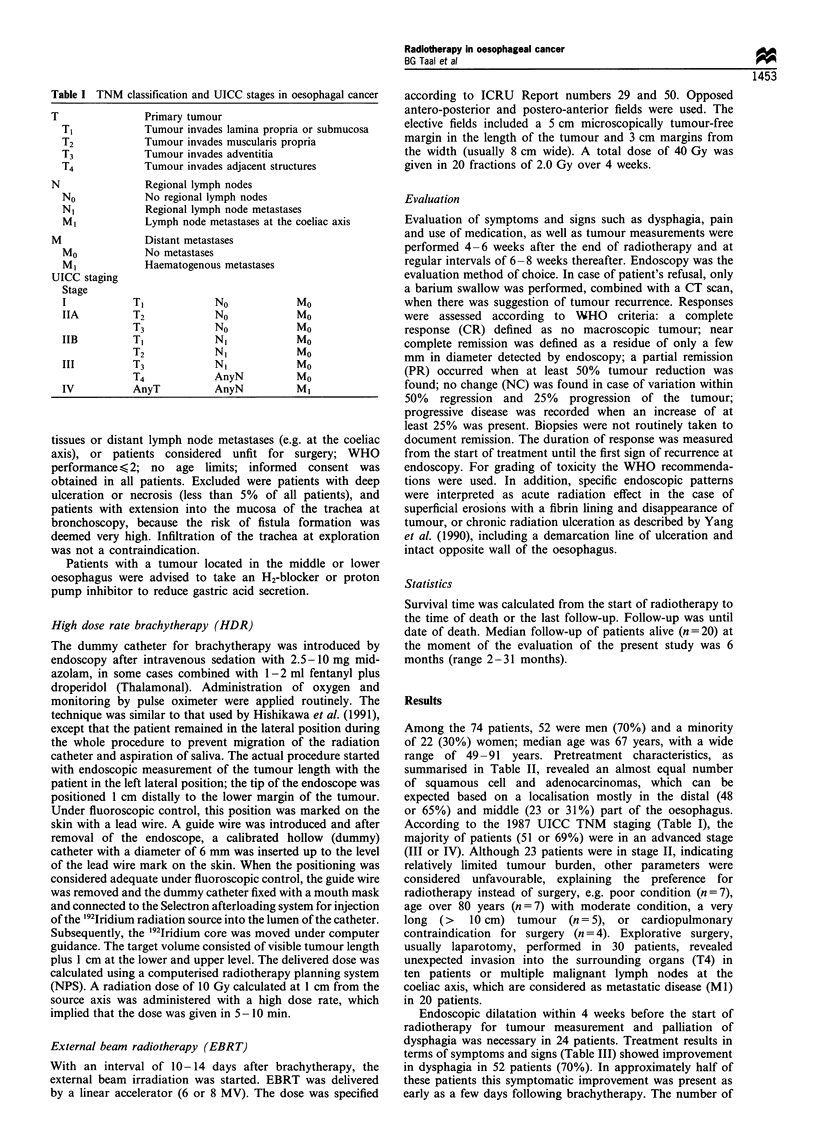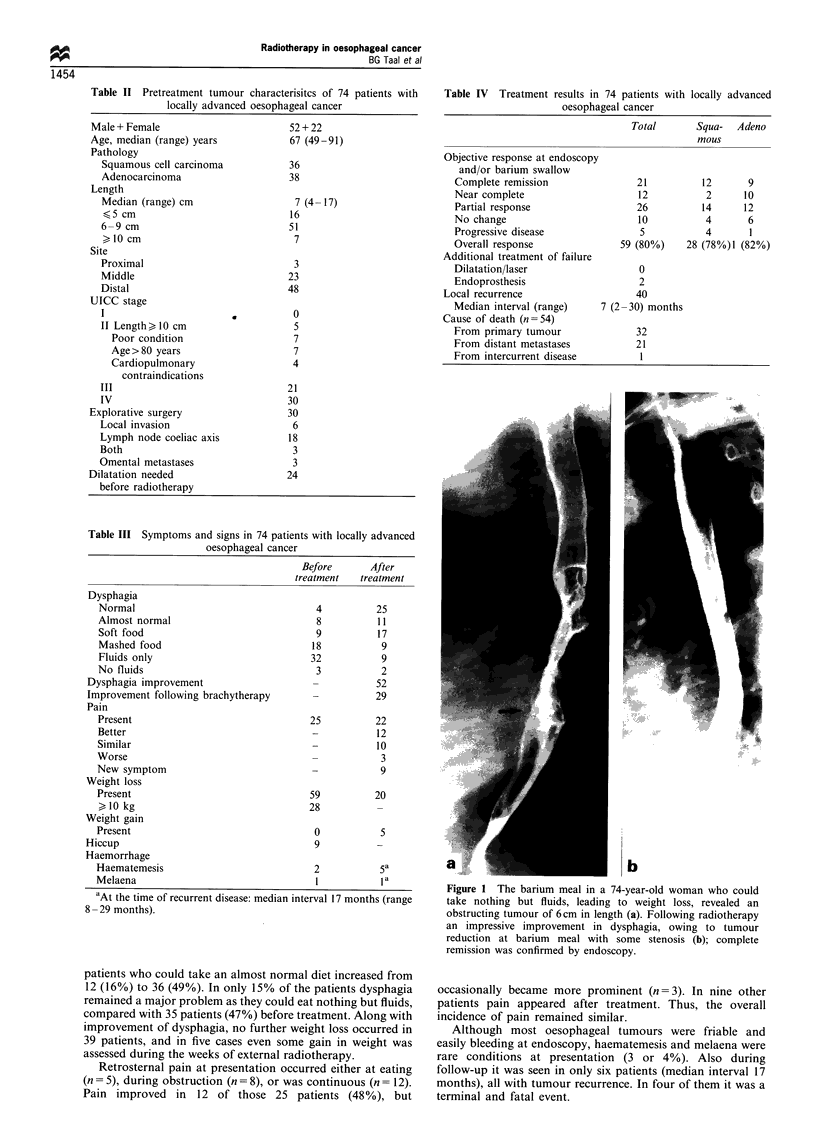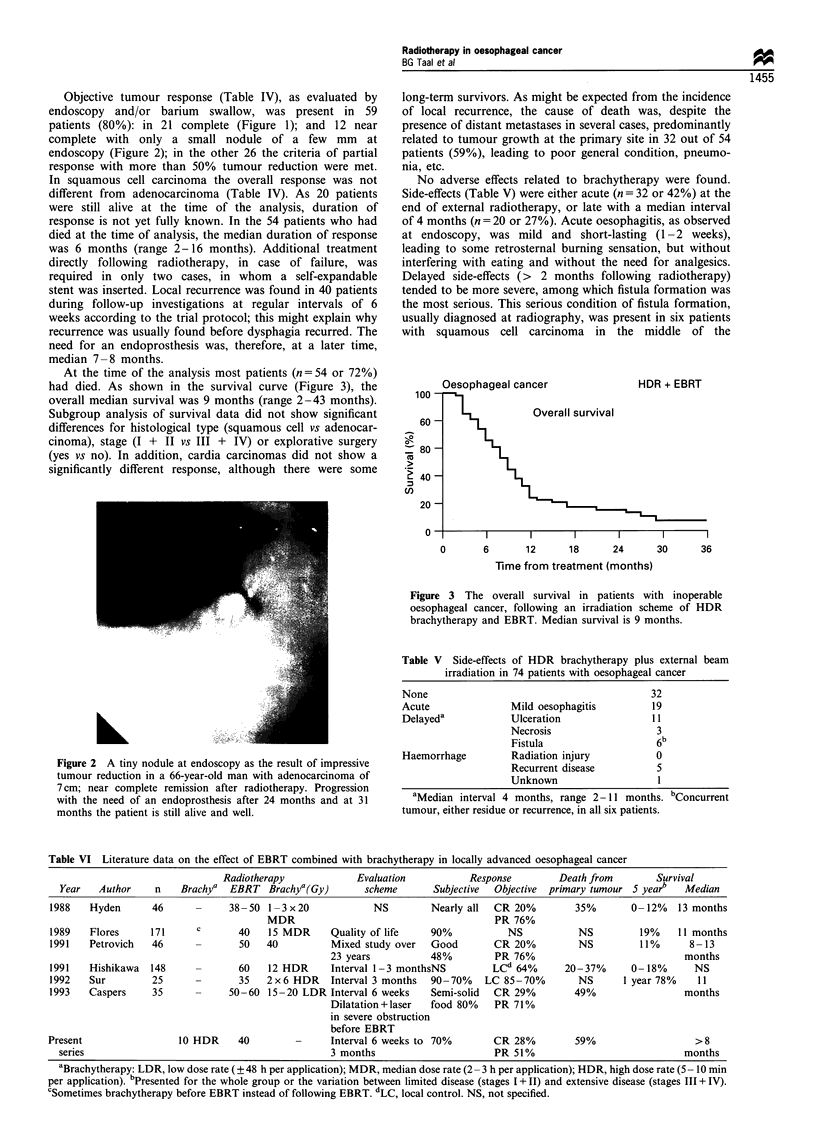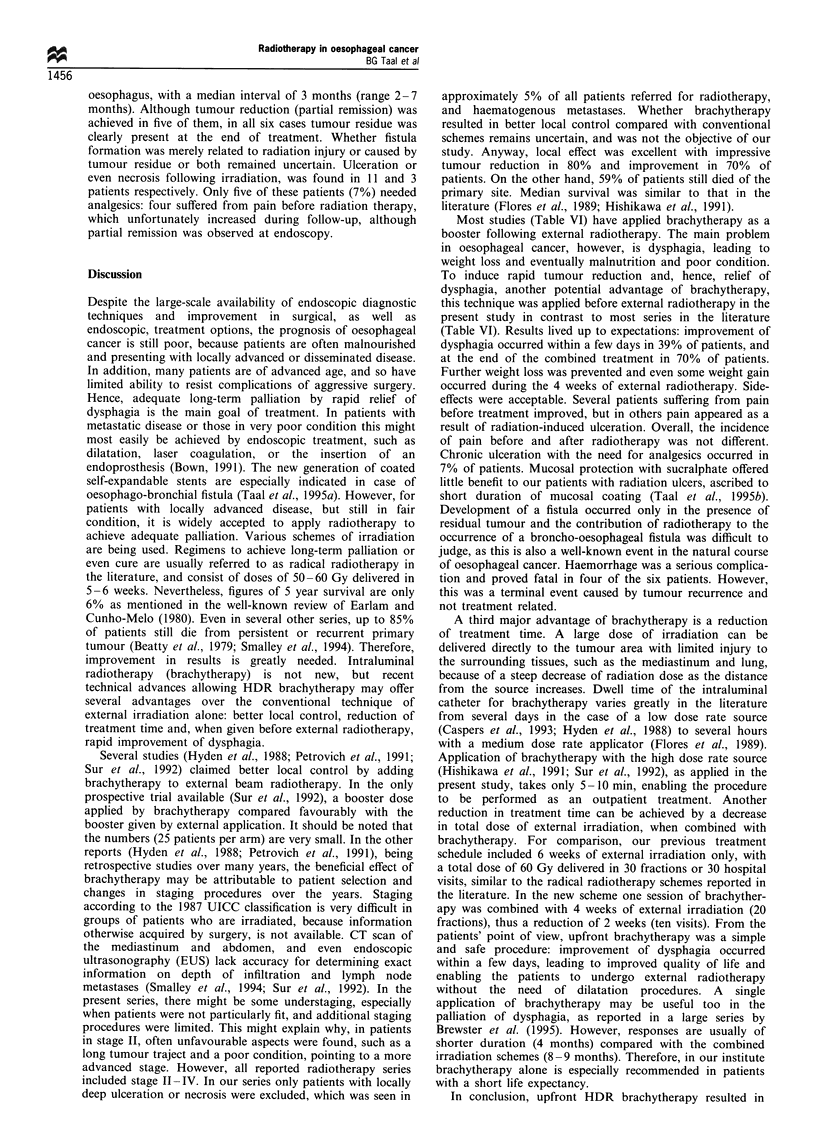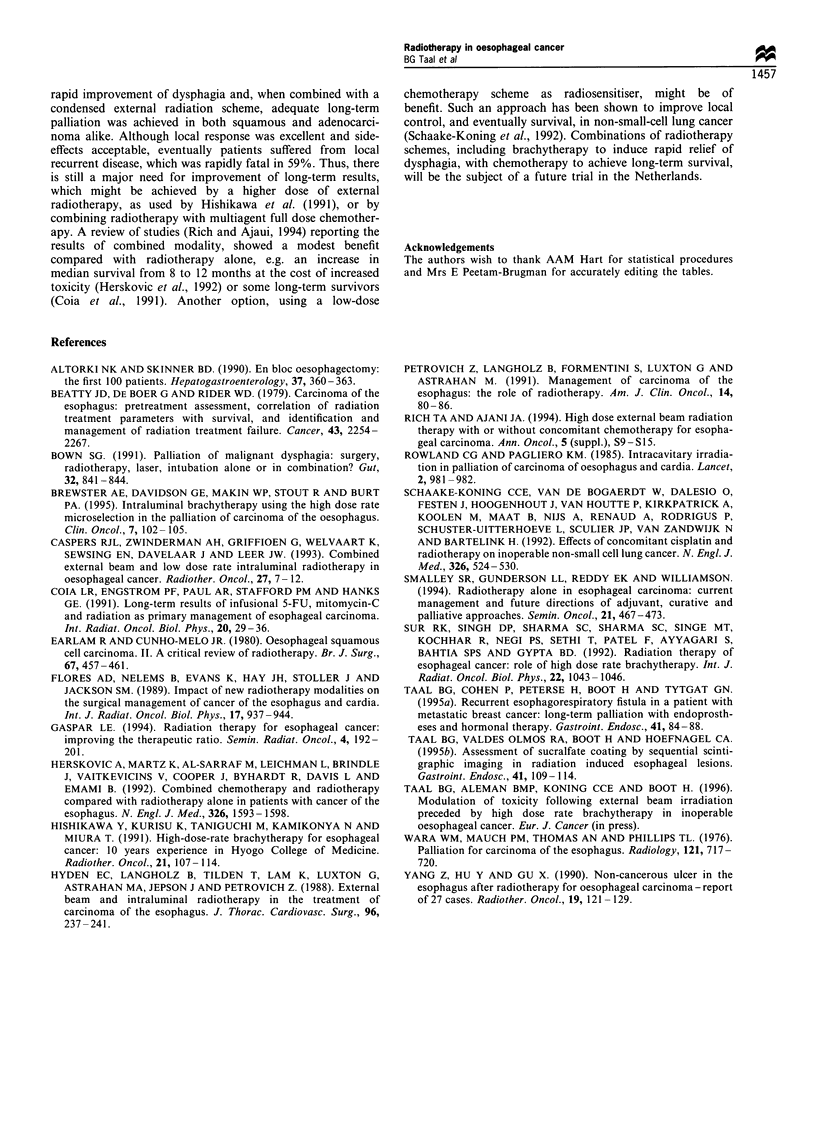# High dose rate brachytherapy before external beam irradiation in inoperable oesophageal cancer.

**DOI:** 10.1038/bjc.1996.564

**Published:** 1996-11

**Authors:** B. G. Taal, B. M. Aleman, C. C. Koning, H. Boot

**Affiliations:** Department of Gastroenterology, Netherlands Cancer Institute/Antoni van Leeuwenhoekhuis, Amsterdam, The Netherlands.

## Abstract

**Images:**


British Journal of Cancer (1996) 74, 1452-1457
© 1996 Stockton Press All rights reserved 0007-0920/96 $12.00

### High dose rate brachytherapy before external beam irradiation in inoperable oesophageal cancer

BG Taal[1], BMP Aleman[2], CCE Koning[2] and H Boot[1]

*Departments of [1]Gastroenterology and [2]Radiotherapy, Netherlands Cancer Institute/Antoni van Leeuwenhoekhuis, Amsterdam, The Netherlands.*

**Summary** To induce fast relief of dysphagia in patients with oesophageal cancer high dose rate (HDR) brachytherapy was applied before external radiotherapy in a prospective study. Seventy-four patients with inoperable oesophageal cancer (36 squamous cell, 38 adenocarcinoma) were treated with a combination of 10 Gy HDR brachytherapy, followed by 40 Gy in 4 weeks external beam radiotherapy (EBRT), starting 2 weeks later. Tumour response, as measured by endoscopy and/or barium swallow, revealed complete remission in 21 and partial response in 38 patients (overall response rate 80%). Improvement of dysphagia was induced by brachytherapy within a few days in 39%, and achieved at the end of treatment in 70% of patients. Further weight loss was prevented in 39 of the 59 patients who presented with weight loss. Pain at presentation improved in 12 out of 25 patients. Median survival was 9 months. No differences in either response rate or survival were found in squamous cell or adenocarcinoma. Side-effects were either acute with minimal discomfort in 32 (42%) or late with painful ulceration in five patients (7%), occurring after a median of 4 months. A fistula developed in six patients, all with concurrent tumour. In conclusion, brachytherapy before EBRT was a safe and effective procedure to induce rapid relief of dysphagia, especially when combined with EBRT.

**Keywords:** oesophageal cancer; brachytherapy; radiotherapy

The prognosis of oesophageal cancer is usually dismal. Surgery is potentially curative in only a small subset of patients (Altorki and Skinner, 1990). Oesophageal resection is considered major surgery, which includes substantial morbidity and mortality. Although post-operative mortality has decreased over the last years and is acceptably low (5%) in some centres, resection as a palliative measure is not widely accepted in clinical practice. In the case of unresectable oesophageal cancer or advanced locoregional disease, radical radiotherapy may offer adequate palliation (Beatty *et al.*, 1979), although overall prognosis is poor and progression may be apparent even during irradiation in up to 20% of patients (Wara *et al.*, 1976). Since the review of the literature (1954-1979) on the role of radiotherapy by Earlam and Cunho-Melo (1980), radiation techniques have changed, especially after brachytherapy was in favour again when remote control techniques became available, as described by Rowland and Pagliero (1985); followed by others (Petrovich *et al.*, 1991; Smalley *et al.*, 1994). In most studies (Caspers *et al.*, 1993; Flores *et al.*, 1989; Gaspar, 1994; Hishikawa *et al.*, 1991; Hyden *et al.*, 1988; Sur *et al.*, 1992), intracavitary irradiation has been used as a booster following external radiotherapy, which has the advantage of delivering a high dose to a small tumour volume.

As dysphagia is the main symptom in oesophageal cancer leading to weight loss and deterioration of the general condition, we applied brachytherapy before EBRT to induce rapid tumour reduction and subsequent relief of dysphagia. Aiming at optimal tumour regression and improved quality of life, at the cost of minimal side-effects, we based our regimen on the two largest series in the literature: that of Flores *et al.* (1989), who combined a short course of EBRT (40 Gy over 3 weeks) with medium dose rate brachytherapy (15 Gy), and the wide experience of Hishikawa *et al.* (1991) using high dose rate (2 × 6 Gy) in addition to EBRT (60 Gy over 6 weeks). With regard to dysphagia and the generally short life expectancy, we applied 10 Gy HDR brachytherapy before a short duration radiotherapy scheme, 40 Gy over 3 weeks EBRT. When this appeared to be too toxic in a pilot study, in nine out of 15 patients severe late side-effects, e.g. ulceration, necrosis and fistula formation (Taal *et al.*, 1996), we adapted the regimen by giving the same EBRT dose over 4 weeks. In comparison, in the past, when brachytherapy was not yet available, we applied a 6 week scheme of EBRT only: 40 Gy over 4 weeks plus 20 Gy over 2 weeks as a booster dose to a smaller volume.

In this paper we report on response and side-effects of our new scheme with upfront brachytherapy in a prospective phase II trial.

### Materials and methods

### *Patients*

Between February 1991 and September 1994, 74 consecutive patients with advanced inoperable oesophageal cancer entered a prospective study of radiotherapy at the Netherlands Cancer Institute approved by the local medical ethics committee. All patients, including those from the referring hospitals, underwent a diagnostic endoscopy in our institute and were discussed by the gastroenterologist and radiation oncologist. The diagnosis was based on endoscopic biopsies. Routine staging procedures consisted of physical examination, laboratory tests (haematology and blood chemistry), chest radiograph, a barium swallow, endoscopy, computerised tomography (CT) scan of the mediastinum and liver or ultrasonography of the liver. In addition to this clinical staging, information available from explorative laparotomy was included in 30 patients. The extent of disease was classified according to the 1987 UICC staging system using the TNM classification (Table I). Because this staging system is especially developed for surgically treated patients, additionally other tumour characteristics are mentioned. Still, there is some 'understaging' in patients who did not undergo surgery.

Eligibility criteria included: adeno- or squamous cell carcinoma of the oesophagus or cardiac junction when the main part of the tumour was localised in the oesophagus, inoperable tumours owing to infiltration into surrounding

Correspondence: BG Taal, Nederlands Kanker Instituut/Antoni van Leeuwenhoekhuis, Plesmanlaan 121, 1066 CX Amsterdam, The Netherlands
Received 15 January 1996; revised 21 May 1996; accepted 24 May 1996

**Table I** TNM classification and UICC stages in oesophagal cancer

| | | | |
|---|---|---|---|
| T | Primary tumour | | |
| $T_1$ | Tumour invades lamina propria or submucosa | | |
| $T_2$ | Tumour invades muscularis propria | | |
| $T_3$ | Tumour invades adventitia | | |
| $T_4$ | Tumour invades adjacent structures | | |
| N | Regional lymph nodes | | |
| $N_0$ | No regional lymph nodes | | |
| $N_1$ | Regional lymph node metastases | | |
| $M_1$ | Lymph node metastases at the coeliac axis | | |
| M | Distant metastases | | |
| $M_0$ | No metastases | | |
| $M_1$ | Haematogenous metastases | | |
| UICC staging | | | |
| Stage | | | |
| I | $T_1$ | $N_0$ | $M_0$ |
| IIA | $T_2$ | $N_0$ | $M_0$ |
| | $T_3$ | $N_0$ | $M_0$ |
| IIB | $T_1$ | $N_1$ | $M_0$ |
| | $T_2$ | $N_1$ | $M_0$ |
| III | $T_3$ | $N_1$ | $M_0$ |
| | $T_4$ | AnyN | $M_0$ |
| IV | AnyT | AnyN | $M_1$ |

tissues or distant lymph node metastases (e.g. at the coeliac axis), or patients considered unfit for surgery; WHO performance$\leqslant$2; no age limits; informed consent was obtained in all patients. Excluded were patients with deep ulceration or necrosis (less than 5% of all patients), and patients with extension into the mucosa of the trachea at bronchoscopy, because the risk of fistula formation was deemed very high. Infiltration of the trachea at exploration was not a contraindication.

Patients with a tumour located in the middle or lower oesophagus were advised to take an $H_2$-blocker or proton pump inhibitor to reduce gastric acid secretion.

### *High dose rate brachytherapy (HDR)*

The dummy catheter for brachytherapy was introduced by endoscopy after intravenous sedation with 2.5–10 mg midazolam, in some cases combined with 1–2 ml fentanyl plus droperidol (Thalamonal). Administration of oxygen and monitoring by pulse oximeter were applied routinely. The technique was similar to that used by Hishikawa *et al.* (1991), except that the patient remained in the lateral position during the whole procedure to prevent migration of the radiation catheter and aspiration of saliva. The actual procedure started with endoscopic measurement of the tumour length with the patient in the left lateral position; the tip of the endoscope was positioned 1 cm distally to the lower margin of the tumour. Under fluoroscopic control, this position was marked on the skin with a lead wire. A guide wire was introduced and after removal of the endoscope, a calibrated hollow (dummy) catheter with a diameter of 6 mm was inserted up to the level of the lead wire mark on the skin. When the positioning was considered adequate under fluoroscopic control, the guide wire was removed and the dummy catheter fixed with a mouth mask and connected to the Selectron afterloading system for injection of the $^{192}$Iridium radiation source into the lumen of the catheter. Subsequently, the $^{192}$Iridium core was moved under computer guidance. The target volume consisted of visible tumour length plus 1 cm at the lower and upper level. The delivered dose was calculated using a computerised radiotherapy planning system (NPS). A radiation dose of 10 Gy calculated at 1 cm from the source axis was administered with a high dose rate, which implied that the dose was given in 5–10 min.

### *External beam radiotherapy (EBRT)*

With an interval of 10–14 days after brachytherapy, the external beam irradiation was started. EBRT was delivered by a linear accelerator (6 or 8 MV). The dose was specified according to ICRU Report numbers 29 and 50. Opposed antero-posterior and postero-anterior fields were used. The elective fields included a 5 cm microscopically tumour-free margin in the length of the tumour and 3 cm margins from the width (usually 8 cm wide). A total dose of 40 Gy was given in 20 fractions of 2.0 Gy over 4 weeks.

### *Evaluation*

Evaluation of symptoms and signs such as dysphagia, pain and use of medication, as well as tumour measurements were performed 4–6 weeks after the end of radiotherapy and at regular intervals of 6–8 weeks thereafter. Endoscopy was the evaluation method of choice. In case of patient's refusal, only a barium swallow was performed, combined with a CT scan, when there was suggestion of tumour recurrence. Responses were assessed according to WHO criteria: a complete response (CR) defined as no macroscopic tumour; near complete remission was defined as a residue of only a few mm in diameter detected by endoscopy; a partial remission (PR) occurred when at least 50% tumour reduction was found; no change (NC) was found in case of variation within 50% regression and 25% progression of the tumour; progressive disease was recorded when an increase of at least 25% was present. Biopsies were not routinely taken to document remission. The duration of response was measured from the start of treatment until the first sign of recurrence at endoscopy. For grading of toxicity the WHO recommendations were used. In addition, specific endoscopic patterns were interpreted as acute radiation effect in the case of superficial erosions with a fibrin lining and disappearance of tumour, or chronic radiation ulceration as described by Yang *et al.* (1990), including a demarcation line of ulceration and intact opposite wall of the oesophagus.

### *Statistics*

Survival time was calculated from the start of radiotherapy to the time of death or the last follow-up. Follow-up was until date of death. Median follow-up of patients alive ($n$=20) at the moment of the evaluation of the present study was 6 months (range 2–31 months).

### Results

Among the 74 patients, 52 were men (70%) and a minority of 22 (30%) women; median age was 67 years, with a wide range of 49–91 years. Pretreatment characteristics, as summarised in Table II, revealed an almost equal number of squamous cell and adenocarcinomas, which can be expected based on a localisation mostly in the distal (48 or 65%) and middle (23 or 31%) part of the oesophagus. According to the 1987 UICC TNM staging (Table I), the majority of patients (51 or 69%) were in an advanced stage (III or IV). Although 23 patients were in stage II, indicating relatively limited tumour burden, other parameters were considered unfavourable, explaining the preference for radiotherapy instead of surgery, e.g. poor condition ($n$=7), age over 80 years ($n$=7) with moderate condition, a very long (> 10 cm) tumour ($n$=5), or cardiopulmonary contraindication for surgery ($n$=4). Explorative surgery, usually laparotomy, performed in 30 patients, revealed unexpected invasion into the surrounding organs (T4) in ten patients or multiple malignant lymph nodes at the coeliac axis, which are considered as metastatic disease (M1) in 20 patients.

Endoscopic dilatation within 4 weeks before the start of radiotherapy for tumour measurement and palliation of dysphagia was necessary in 24 patients. Treatment results in terms of symptoms and signs (Table III) showed improvement in dysphagia in 52 patients (70%). In approximately half of these patients this symptomatic improvement was present as early as a few days following brachytherapy. The number of

**Table II** Pretreatment tumour characterisitcs of 74 patients with locally advanced oesophageal cancer

| | |
|---|---|
| Male + Female | 52 + 22 |
| Age, median (range) years | 67 (49–91) |
| Pathology | |
| Squamous cell carcinoma | 36 |
| Adenocarcinoma | 38 |
| Length | |
| Median (range) cm | 7 (4–17) |
| ⩽5 cm | 16 |
| 6–9 cm | 51 |
| ⩾10 cm | 7 |
| Site | |
| Proximal | 3 |
| Middle | 23 |
| Distal | 48 |
| UICC stage | |
| I | 0 |
| II Length ⩾ 10 cm | 5 |
| Poor condition | 7 |
| Age > 80 years | 7 |
| Cardiopulmonary contraindications | 4 |
| III | 21 |
| IV | 30 |
| Explorative surgery | 30 |
| Local invasion | 6 |
| Lymph node coeliac axis | 18 |
| Both | 3 |
| Omental metastases | 3 |
| Dilatation needed before radiotherapy | 24 |

**Table III** Symptoms and signs in 74 patients with locally advanced oesophageal cancer

| | *Before treatment* | *After treatment* |
|---|---|---|
| Dysphagia | | |
| Normal | 4 | 25 |
| Almost normal | 8 | 11 |
| Soft food | 9 | 17 |
| Mashed food | 18 | 9 |
| Fluids only | 32 | 9 |
| No fluids | 3 | 2 |
| Dysphagia improvement | – | 52 |
| Improvement following brachytherapy | – | 29 |
| Pain | | |
| Present | 25 | 22 |
| Better | – | 12 |
| Similar | – | 10 |
| Worse | – | 3 |
| New symptom | – | 9 |
| Weight loss | | |
| Present | 59 | 20 |
| ⩾10 kg | 28 | – |
| Weight gain | | |
| Present | 0 | 5 |
| Hiccup | 9 | – |
| Haemorrhage | | |
| Haematemesis | 2 | 5[a] |
| Melaena | 1 | 1[a] |

[a]At the time of recurrent disease: median interval 17 months (range 8–29 months).

**Table IV** Treatment results in 74 patients with locally advanced oesophageal cancer

| | *Total* | *Squamous* | *Adeno* |
|---|---|---|---|
| Objective response at endoscopy and/or barium swallow | | | |
| Complete remission | 21 | 12 | 9 |
| Near complete | 12 | 2 | 10 |
| Partial response | 26 | 14 | 12 |
| No change | 10 | 4 | 6 |
| Progressive disease | 5 | 4 | 1 |
| Overall response | 59 (80%) | 28 (78%) | 1 (82%) |
| Additional treatment of failure | | | |
| Dilatation/laser | 0 | | |
| Endoprosthesis | 2 | | |
| Local recurrence | 40 | | |
| Median interval (range) | 7 (2–30) months | | |
| Cause of death ($n = 54$) | | | |
| From primary tumour | 32 | | |
| From distant metastases | 21 | | |
| From intercurrent disease | 1 | | |

**Figure 1** The barium meal in a 74-year-old woman who could take nothing but fluids, leading to weight loss, revealed an obstructing tumour of 6 cm in length (**a**). Following radiotherapy an impressive improvement in dysphagia, owing to tumour reduction at barium meal with some stenosis (**b**); complete remission was confirmed by endoscopy.

patients who could take an almost normal diet increased from 12 (16%) to 36 (49%). In only 15% of the patients dysphagia remained a major problem as they could eat nothing but fluids, compared with 35 patients (47%) before treatment. Along with improvement of dysphagia, no further weight loss occurred in 39 patients, and in five cases even some gain in weight was assessed during the weeks of external radiotherapy.

Retrosternal pain at presentation occurred either at eating ($n = 5$), during obstruction ($n = 8$), or was continuous ($n = 12$). Pain improved in 12 of those 25 patients (48%), but occasionally became more prominent ($n = 3$). In nine other patients pain appeared after treatment. Thus, the overall incidence of pain remained similar.

Although most oesophageal tumours were friable and easily bleeding at endoscopy, haematemesis and melaena were rare conditions at presentation (3 or 4%). Also during follow-up it was seen in only six patients (median interval 17 months), all with tumour recurrence. In four of them it was a terminal and fatal event.

Objective tumour response (Table IV), as evaluated by endoscopy and/or barium swallow, was present in 59 patients (80%): in 21 complete (Figure 1); and 12 near complete with only a small nodule of a few mm at endoscopy (Figure 2); in the other 26 the criteria of partial response with more than 50% tumour reduction were met. In squamous cell carcinoma the overall response was not different from adenocarcinoma (Table IV). As 20 patients were still alive at the time of the analysis, duration of response is not yet fully known. In the 54 patients who had died at the time of analysis, the median duration of response was 6 months (range 2–16 months). Additional treatment directly following radiotherapy, in case of failure, was required in only two cases, in whom a self-expandable stent was inserted. Local recurrence was found in 40 patients during follow-up investigations at regular intervals of 6 weeks according to the trial protocol; this might explain why recurrence was usually found before dysphagia recurred. The need for an endoprosthesis was, therefore, at a later time, median 7–8 months.

At the time of the analysis most patients ($n=54$ or 72%) had died. As shown in the survival curve (Figure 3), the overall median survival was 9 months (range 2–43 months). Subgroup analysis of survival data did not show significant differences for histological type (squamous cell *vs* adenocarcinoma), stage (I + II *vs* III + IV) or explorative surgery (yes *vs* no). In addition, cardia carcinomas did not show a significantly different response, although there were some long-term survivors. As might be expected from the incidence of local recurrence, the cause of death was, despite the presence of distant metastases in several cases, predominantly related to tumour growth at the primary site in 32 out of 54 patients (59%), leading to poor general condition, pneumonia, etc.

No adverse effects related to brachytherapy were found. Side-effects (Table V) were either acute ($n=32$ or 42%) at the end of external radiotherapy, or late with a median interval of 4 months ($n=20$ or 27%). Acute oesophagitis, as observed at endoscopy, was mild and short-lasting (1–2 weeks), leading to some retrosternal burning sensation, but without interfering with eating and without the need for analgesics. Delayed side-effects (> 2 months following radiotherapy) tended to be more severe, among which fistula formation was the most serious. This serious condition of fistula formation, usually diagnosed at radiography, was present in six patients with squamous cell carcinoma in the middle of the

**Figure 2** A tiny nodule at endoscopy as the result of impressive tumour reduction in a 66-year-old man with adenocarcinoma of 7 cm; near complete remission after radiotherapy. Progression with the need of an endoprosthesis after 24 months and at 31 months the patient is still alive and well.

**Figure 3** The overall survival in patients with inoperable oesophageal cancer, following an irradiation scheme of HDR brachytherapy and EBRT. Median survival is 9 months.

**Table V** Side-effects of HDR brachytherapy plus external beam irradiation in 74 patients with oesophageal cancer

| | | |
|---|---|---|
| None | | 32 |
| Acute | Mild oesophagitis | 19 |
| Delayed[a] | Ulceration | 11 |
| | Necrosis | 3 |
| | Fistula | 6[b] |
| Haemorrhage | Radiation injury | 0 |
| | Recurrent disease | 5 |
| | Unknown | 1 |

[a]Median interval 4 months, range 2–11 months. [b]Concurrent tumour, either residue or recurrence, in all six patients.

**Table VI** Literature data on the effect of EBRT combined with brachytherapy in locally advanced oesophageal cancer

| | | | *Radiotherapy* | | | *Evaluation* | *Response* | | *Death from* | *Survival* | |
|---|---|---|---|---|---|---|---|---|---|---|---|
| *Year* | *Author* | n | *Brachy*[a] | *EBRT* | *Brachy*[a] *(Gy)* | *scheme* | *Subjective* | *Objective* | *primary tumour* | *5 year*[b] | *Median* |
| 1988 | Hyden | 46 | – | 38–50 | 1–3 × 20 MDR | NS | Nearly all | CR 20% PR 76% | 35% | 0–12% | 13 months |
| 1989 | Flores | 171 | [c] | 40 | 15 MDR | Quality of life | 90% | NS | NS | 19% | 11 months |
| 1991 | Petrovich | 46 | – | 50 | 40 | Mixed study over 23 years | Good 48% | CR 20% PR 76% | NS | 11% | 8–13 months |
| 1991 | Hishikawa | 148 | – | 60 | 12 HDR | Interval 1–3 months | NS | LC[d] 64% | 20–37% | 0–18% | NS |
| 1992 | Sur | 25 | – | 35 | 2 × 6 HDR | Interval 3 months | 90–70% | LC 85–70% | NS | 1 year 78% | 11 |
| 1993 | Caspers | 35 | – | 50–60 | 15–20 LDR | Interval 6 weeks Dilatation + laser in severe obstruction before EBRT | Semi-solid food 80% | CR 29% PR 71% | 49% | | months |
| Present series | | | 10 HDR | 40 | – | Interval 6 weeks to 3 months | 70% | CR 28% PR 51% | 59% | | >8 months |

[a]Brachytherapy: LDR, low dose rate (±48 h per application); MDR, median dose rate (2–3 h per application); HDR, high dose rate (5–10 min per application). [b]Presented for the whole group or the variation between limited disease (stages I + II) and extensive disease (stages III + IV). [c]Sometimes brachytherapy before EBRT instead of following EBRT. [d]LC, local control. NS, not specified.

oesophagus, with a median interval of 3 months (range 2–7 months). Although tumour reduction (partial remission) was achieved in five of them, in all six cases tumour residue was clearly present at the end of treatment. Whether fistula formation was merely related to radiation injury or caused by tumour residue or both remained uncertain. Ulceration or even necrosis following irradiation, was found in 11 and 3 patients respectively. Only five of these patients (7%) needed analgesics: four suffered from pain before radiation therapy, which unfortunately increased during follow-up, although partial remission was observed at endoscopy.

### Discussion

Despite the large-scale availability of endoscopic diagnostic techniques and improvement in surgical, as well as endoscopic, treatment options, the prognosis of oesophageal cancer is still poor, because patients are often malnourished and presenting with locally advanced or disseminated disease. In addition, many patients are of advanced age, and so have limited ability to resist complications of aggressive surgery. Hence, adequate long-term palliation by rapid relief of dysphagia is the main goal of treatment. In patients with metastatic disease or those in very poor condition this might most easily be achieved by endoscopic treatment, such as dilatation, laser coagulation, or the insertion of an endoprosthesis (Bown, 1991). The new generation of coated self-expandable stents are especially indicated in case of oesophago-bronchial fistula (Taal *et al.*, 1995*a*). However, for patients with locally advanced disease, but still in fair condition, it is widely accepted to apply radiotherapy to achieve adequate palliation. Various schemes of irradiation are being used. Regimens to achieve long-term palliation or even cure are usually referred to as radical radiotherapy in the literature, and consist of doses of 50–60 Gy delivered in 5–6 weeks. Nevertheless, figures of 5 year survival are only 6% as mentioned in the well-known review of Earlam and Cunho-Melo (1980). Even in several other series, up to 85% of patients still die from persistent or recurrent primary tumour (Beatty *et al.*, 1979; Smalley *et al.*, 1994). Therefore, improvement in results is greatly needed. Intraluminal radiotherapy (brachytherapy) is not new, but recent technical advances allowing HDR brachytherapy may offer several advantages over the conventional technique of external irradiation alone: better local control, reduction of treatment time and, when given before external radiotherapy, rapid improvement of dysphagia.

Several studies (Hyden *et al.*, 1988; Petrovich *et al.*, 1991; Sur *et al.*, 1992) claimed better local control by adding brachytherapy to external beam radiotherapy. In the only prospective trial available (Sur *et al.*, 1992), a booster dose applied by brachytherapy compared favourably with the booster given by external application. It should be noted that the numbers (25 patients per arm) are very small. In the other reports (Hyden *et al.*, 1988; Petrovich *et al.*, 1991), being retrospective studies over many years, the beneficial effect of brachytherapy may be attributable to patient selection and changes in staging procedures over the years. Staging according to the 1987 UICC classification is very difficult in groups of patients who are irradiated, because information otherwise acquired by surgery, is not available. CT scan of the mediastinum and abdomen, and even endoscopic ultrasonography (EUS) lack accuracy for determining exact information on depth of infiltration and lymph node metastases (Smalley *et al.*, 1994; Sur *et al.*, 1992). In the present series, there might be some understaging, especially when patients were not particularly fit, and additional staging procedures were limited. This might explain why, in patients in stage II, often unfavourable aspects were found, such as a long tumour traject and a poor condition, pointing to a more advanced stage. However, all reported radiotherapy series included stage II–IV. In our series only patients with locally deep ulceration or necrosis were excluded, which was seen in approximately 5% of all patients referred for radiotherapy, and haematogenous metastases. Whether brachytherapy resulted in better local control compared with conventional schemes remains uncertain, and was not the objective of our study. Anyway, local effect was excellent with impressive tumour reduction in 80% and improvement in 70% of patients. On the other hand, 59% of patients still died of the primary site. Median survival was similar to that in the literature (Flores *et al.*, 1989; Hishikawa *et al.*, 1991).

Most studies (Table VI) have applied brachytherapy as a booster following external radiotherapy. The main problem in oesophageal cancer, however, is dysphagia, leading to weight loss and eventually malnutrition and poor condition. To induce rapid tumour reduction and, hence, relief of dysphagia, another potential advantage of brachytherapy, this technique was applied before external radiotherapy in the present study in contrast to most series in the literature (Table VI). Results lived up to expectations: improvement of dysphagia occurred within a few days in 39% of patients, and at the end of the combined treatment in 70% of patients. Further weight loss was prevented and even some weight gain occurred during the 4 weeks of external radiotherapy. Side-effects were acceptable. Several patients suffering from pain before treatment improved, but in others pain appeared as a result of radiation-induced ulceration. Overall, the incidence of pain before and after radiotherapy was not different. Chronic ulceration with the need for analgesics occurred in 7% of patients. Mucosal protection with sucralphate offered little benefit to our patients with radiation ulcers, ascribed to short duration of mucosal coating (Taal *et al.*, 1995*b*). Development of a fistula occurred only in the presence of residual tumour and the contribution of radiotherapy to the occurrence of a broncho-oesophageal fistula was difficult to judge, as this is also a well-known event in the natural course of oesophageal cancer. Haemorrhage was a serious complication and proved fatal in four of the six patients. However, this was a terminal event caused by tumour recurrence and not treatment related.

A third major advantage of brachytherapy is a reduction of treatment time. A large dose of irradiation can be delivered directly to the tumour area with limited injury to the surrounding tissues, such as the mediastinum and lung, because of a steep decrease of radiation dose as the distance from the source increases. Dwell time of the intraluminal catheter for brachytherapy varies greatly in the literature from several days in the case of a low dose rate source (Caspers *et al.*, 1993; Hyden *et al.*, 1988) to several hours with a medium dose rate applicator (Flores *et al.*, 1989). Application of brachytherapy with the high dose rate source (Hishikawa *et al.*, 1991; Sur *et al.*, 1992), as applied in the present study, takes only 5–10 min, enabling the procedure to be performed as an outpatient treatment. Another reduction in treatment time can be achieved by a decrease in total dose of external irradiation, when combined with brachytherapy. For comparison, our previous treatment schedule included 6 weeks of external irradiation only, with a total dose of 60 Gy delivered in 30 fractions or 30 hospital visits, similar to the radical radiotherapy schemes reported in the literature. In the new scheme one session of brachytherapy was combined with 4 weeks of external irradiation (20 fractions), thus a reduction of 2 weeks (ten visits). From the patients' point of view, upfront brachytherapy was a simple and safe procedure: improvement of dysphagia occurred within a few days, leading to improved quality of life and enabling the patients to undergo external radiotherapy without the need of dilatation procedures. A single application of brachytherapy may be useful too in the palliation of dysphagia, as reported in a large series by Brewster *et al.* (1995). However, responses are usually of shorter duration (4 months) compared with the combined irradiation schemes (8–9 months). Therefore, in our institute brachytherapy alone is especially recommended in patients with a short life expectancy.

In conclusion, upfront HDR brachytherapy resulted in rapid improvement of dysphagia and, when combined with a condensed external radiation scheme, adequate long-term palliation was achieved in both squamous and adenocarcinoma alike. Although local response was excellent and side-effects acceptable, eventually patients suffered from local recurrent disease, which was rapidly fatal in 59%. Thus, there is still a major need for improvement of long-term results, which might be achieved by a higher dose of external radiotherapy, as used by Hishikawa *et al.* (1991), or by combining radiotherapy with multiagent full dose chemotherapy. A review of studies (Rich and Ajaui, 1994) reporting the results of combined modality, showed a modest benefit compared with radiotherapy alone, e.g. an increase in median survival from 8 to 12 months at the cost of increased toxicity (Herskovic *et al.*, 1992) or some long-term survivors (Coia *et al.*, 1991). Another option, using a low-dose chemotherapy scheme as radiosensitiser, might be of benefit. Such an approach has been shown to improve local control, and eventually survival, in non-small-cell lung cancer (Schaake-Koning *et al.*, 1992). Combinations of radiotherapy schemes, including brachytherapy to induce rapid relief of dysphagia, with chemotherapy to achieve long-term survival, will be the subject of a future trial in the Netherlands.

**Acknowledgements**
The authors wish to thank AAM Hart for statistical procedures and Mrs E Peetam-Brugman for accurately editing the tables.

### References

ALTORKI NK AND SKINNER BD. (1990). En bloc oesophagectomy: the first 100 patients. *Hepatogastroenterology*, **37**, 360-363.

BEATTY JD, DE BOER G AND RIDER WD. (1979). Carcinoma of the esophagus: pretreatment assessment, correlation of radiation treatment parameters with survival, and identification and management of radiation treatment failure. *Cancer*, **43**, 2254-2267.

BOWN SG. (1991). Palliation of malignant dysphagia: surgery, radiotherapy, laser, intubation alone or in combination? *Gut*, **32**, 841-844.

BREWSTER AE, DAVIDSON GE, MAKIN WP, STOUT R AND BURT PA. (1995). Intraluminal brachytherapy using the high dose rate microselection in the palliation of carcinoma of the oesophagus. *Clin. Oncol.*, **7**, 102-105.

CASPERS RJL, ZWINDERMAN AH, GRIFFIOEN G, WELVAART K, SEWSING EN, DAVELAAR J AND LEER JW. (1993). Combined external beam and low dose rate intraluminal radiotherapy in oesophageal cancer. *Radiother. Oncol.*, **27**, 7-12.

COIA LR, ENGSTROM PF, PAUL AR, STAFFORD PM AND HANKS GE. (1991). Long-term results of infusional 5-FU, mitomycin-C and radiation as primary management of esophageal carcinoma. *Int. Radiat. Oncol. Biol. Phys.*, **20**, 29-36.

EARLAM R AND CUNHO-MELO JR. (1980). Oesophageal squamous cell carcinoma. II. A critical review of radiotherapy. *Br. J. Surg.*, **67**, 457-461.

FLORES AD, NELEMS B, EVANS K, HAY JH, STOLLER J AND JACKSON SM. (1989). Impact of new radiotherapy modalities on the surgical management of cancer of the esophagus and cardia. *Int. J. Radiat. Oncol. Biol. Phys.*, **17**, 937-944.

GASPAR LE. (1994). Radiation therapy for esophageal cancer: improving the therapeutic ratio. *Semin. Radiat. Oncol.*, **4**, 192-201.

HERSKOVIC A, MARTZ K, AL-SARRAF M, LEICHMAN L, BRINDLE J, VAITKEVICINS V, COOPER J, BYHARDT R, DAVIS L AND EMAMI B. (1992). Combined chemotherapy and radiotherapy compared with radiotherapy alone in patients with cancer of the esophagus. *N. Engl. J. Med.*, **326**, 1593-1598.

HISHIKAWA Y, KURISU K, TANIGUCHI M, KAMIKONYA N AND MIURA T. (1991). High-dose-rate brachytherapy for esophageal cancer: 10 years experience in Hyogo College of Medicine. *Radiother. Oncol.*, **21**, 107-114.

HYDEN EC, LANGHOLZ B, TILDEN T, LAM K, LUXTON G, ASTRAHAN MA, JEPSON J AND PETROVICH Z. (1988). External beam and intraluminal radiotherapy in the treatment of carcinoma of the esophagus. *J. Thorac. Cardiovasc. Surg.*, **96**, 237-241.

PETROVICH Z, LANGHOLZ B, FORMENTINI S, LUXTON G AND ASTRAHAN M. (1991). Management of carcinoma of the esophagus: the role of radiotherapy. *Am. J. Clin. Oncol.*, **14**, 80-86.

RICH TA AND AJANI JA. (1994). High dose external beam radiation therapy with or without concomitant chemotherapy for esophageal carcinoma. *Ann. Oncol.*, **5** (suppl.), S9-S15.

ROWLAND CG AND PAGLIERO KM. (1985). Intracavitary irradiation in palliation of carcinoma of oesophagus and cardia. *Lancet*, **2**, 981-982.

SCHAAKE-KONING CCE, VAN DE BOGAERDT W, DALESIO O, FESTEN J, HOOGENHOUT J, VAN HOUTTE P, KIRKPATRICK A, KOOLEN M, MAAT B, NIJS A, RENAUD A, RODRIGUS P, SCHUSTER-UITTERHOEVE L, SCULIER JP, VAN ZANDWIJK N AND BARTELINK H. (1992). Effects of concomitant cisplatin and radiotherapy on inoperable non-small cell lung cancer. *N. Engl. J. Med.*, **326**, 524-530.

SMALLEY SR, GUNDERSON LL, REDDY EK AND WILLIAMSON. (1994). Radiotherapy alone in esophageal carcinoma: current management and future directions of adjuvant, curative and palliative approaches. *Semin. Oncol.*, **21**, 467-473.

SUR RK, SINGH DP, SHARMA SC, SHARMA SC, SINGE MT, KOCHHAR R, NEGI PS, SETHI T, PATEL F, AYYAGARI S, BAHTIA SPS AND GYPTA BD. (1992). Radiation therapy of esophageal cancer: role of high dose rate brachytherapy. *Int. J. Radiat. Oncol. Biol. Phys.*, **22**, 1043-1046.

TAAL BG, COHEN P, PETERSE H, BOOT H AND TYTGAT GN. (1995*a*). Recurrent esophagorespiratory fistula in a patient with metastatic breast cancer: long-term palliation with endoprostheses and hormonal therapy. *Gastroint. Endosc.*, **41**, 84-88.

TAAL BG, VALDES OLMOS RA, BOOT H AND HOEFNAGEL CA. (1995*b*). Assessment of sucralfate coating by sequential scintigraphic imaging in radiation induced esophageal lesions. *Gastroint. Endosc.*, **41**, 109-114.

TAAL BG, ALEMAN BMP, KONING CCE AND BOOT H. (1996). Modulation of toxicity following external beam irradiation preceded by high dose rate brachytherapy in inoperable oesophageal cancer. *Eur. J. Cancer* (in press).

WARA WM, MAUCH PM, THOMAS AN AND PHILLIPS TL. (1976). Palliation for carcinoma of the esophagus. *Radiology*, **121**, 717-720.

YANG Z, HU Y AND GU X. (1990). Non-cancerous ulcer in the esophagus after radiotherapy for oesophageal carcinoma - report of 27 cases. *Radiother. Oncol.*, **19**, 121-129.